# Anæsthesia
*Discussion at the Meeting of the Bristol Medico-Chirurgical Society, on 9th April, 1942.


**Published:** 1942

**Authors:** 


### ANÆSTHESIA.*

Mr. Stuart Stock opened the discussion and spoke on the subject of pre-medication, confining his remarks to paraldehyde and sodium soneryl. He regarded paraldehyde as the safest of all. It has no effect on the respiration or the blood pressure, and reflexes are only slightly diminished after operation. Choloform may be administered with confidence after paraldehyde. The patient rarely complains of the smell. Excitement on wakening could be quietened with morphine. It must be remembered that a very few patients show an idiosyncrasy to the drug. Paraldehyde possessed the advantage that it can be given by the nursing staff. It could be given by mouth, intravenously or by rectum. By mouth the dose was a third of the rectal dose, best given in soda water. For intravenous use paraldehyde is dissolved in Ringer's solution with 1 per cent gum acacia added—dose required 0·75 c.cm. per stone of body weight. He had not used pure paraldehyde intravenously. For rectal administration he allowed three-quarters of a drachm per stone emulsified in a mixture consisting of 32 minims of aqueous tincture of orange to 2 ounces of water.

Sodium Soneryl he recommended for producing amnesia in children over two years of age. The dose should be calculated by weight, not by age. He used capsules containing 0.075 grammes of Soneryl, allowing one capsule up to 35 lbs. weight and an additional half-capsule for every additional 10 lbs. of body weight. The capsules were given in jam by mouth with atropin hypodermically. Children tolerate small doses of morphine well, $\frac{1}{32}$ grain up to 45 lbs. weight, $\frac{1}{24}$ up to 55 lbs., $\frac{1}{16}$ up to 65 lbs. and $\frac{1}{12}$ up to 75 lbs.

Dr. E. G. Bradbeer dealt with the instruction of students. He considered open ether was the best method for teaching purposes. Since the student was only required to administer thirty anæsthetics whilst under instruction, he suggested that only one method should be taught and surgeons preferred residents and students to use open ether. If there were objections in particular patients to the use of open ether he would advise the use of the appropriate anæsthetics to suit the case.

He doubted the value of teaching every student several different methods. He would like to see more resident anæsthetist posts with a senior resident anæsthetist having the status of a registrar. It was desirable that anæsthetics should be systematically taught as a post-graduate subject

Dr. A. P. Gorham discussed pre-medication and basal narcosis, in which he was particularly interested. Induction of inhalation anæsthesia was easier in a quiet patient, hence the value of omnopon-scopolamine together with atropin for pre-medication. Residents

* Discussion at the Meeting of the Bristol Medico-Chirurgical Society, on 9th April, 1942.

and sisters should be thoroughly instructed in the methods of pre-medication and basal narcosis.

He agreed with Mr. Stock that paraldehyde was most satisfactory especially for children. The dosage he used was 1 dram. per stone up to 1 oz. dissolved in tap water rectally. Avertin, for which he had a preference, was little used in Bristol, and the war was restricting its use generally on account of shortage of nursing personnel. He had found it admirable for thyrotoxicosis and brain operations in conjunction with nitrous oxide and oxygen.

One effect of avertin was to produce in some individuals a great fall in blood-pressure, hence it should not be used in conjunction with spinal anaesthesia. Coramine was an excellent " antidote " for this fall in blood-pressure. Occasionally a rise in blood-pressure occurred with avertin, this was an idiosyncrasy which should be recognized.

Major Sykes, R.A.M.C., spoke of post-operative pulmonary complications which were more frequent in soldiers than in civilians.

In the course of 150 operations twenty-four per cent. of patients suffered from pulmonary complications during his first few weeks experience in a military hospital. He attributed this to the fact that the average soldier having lived in the open comes into hospital looking fit and on that account sometimes has insufficient pre-operative investigation. Many of them have coughs and colds that are overlooked. When he refused to permit operations on any patients suffering from the slightest cold the post-operative pulmonary complication rate fell from twenty-four to six per cent.

He had seen one striking case of a soldier, operated on for radical cure of hernia under a local anæsthetic, who developed collapse of the lung after the operation. This could not be due to the anæsthetic, he attributed it to a nurse with a cold who was looking after the man. He referred in conclusion to ether convulsions, which, he said, could be stopped at once by pentothal or evipan.

Mr. Wilfred Adams devoted his remarks to spinal anæsthesia, in which he said he endeavoured to follow Pitkin's teaching. He had operated on 1,000 cases using spinocain. Percaine permitted a longer time for an operation and was to be preferred especially in abdominal and more particularly gastric operations.

The patient needs guarding from disturbing stimuli, therefore pre-medication with omnopon-scopolamine was advisable. He also employed local nerve-block. The expert never fails in finding the spinal theca. He admitted that there was a danger of death if the spinal injection was left to amateur hands. The after-effects were sometimes unpleasant. Backache and in a small proportion of cases severe headache were complained of. Paralysis of spinal and cranial nerves sometimes occurred and might persist for a few months. Metabolism was reduced for some hours and the blood-pressure might fall. He would never use spinal anæsthesia on a patient whose blood-pressure was below 110: in shocked cases spinal anæsthesia was always contra-indicated.

Mr. Kindersley believed that post-operative chest complications were due to lack of supervision after the patient returned to the ward. He considered that the Anæsthetist and Surgeon should together decide upon the choice of anæsthetic.

The Surgeon asks that a patient shall be in a state in which he can do his work, therefore there must be relaxation, a good blood-colour and respiration reduced so as not to interfere with the operation. The honorary anæsthetist should teach the resident anæsthetists and not merely look on them as useful deputies in his absence.

Group Captain Macintosh, R.A.F.M.S., said he was impressed by the practical nature of the discussion. As regards pre-medication he gave a warning of the deaths that had occurred from overdosage with paraldehyde. Anæsthetists must make sure that they write down the dosage clearly for each case. Excitement was liable to occur after any basal anæsthetic. This was due to recovery into a stimulable stage, and the remedy was morphine. Pure paraldehyde can be given intravenously. He had noticed that drugs given orally are apt to be irregularly absorbed.

He agreed with Mr. Stock on the giving of morphine to children. It was especially useful when local anæsthesia was used in children. The dosage on which he worked was $\frac{1}{40}$ grain per stone.

He considered that one method was best for teaching; he preferred open ether. In America he had found that the hospitals specialized on one method, but the method was different in each hospital. From which he concluded that the factor of real safety was that the anæsthetist was well taught and well practised in the method he employed.

Anæsthetics in private and in hospital were different. In private all the induction was complete before the patient was moved from bed. In hospital the whole process of induction must be demonstrated, therefore the patient is taken to the theatre awake.

Skilled nursing was an essential part of basal anæsthesia.

As regards post-operative chest complications he agreed that no operation should be performed on a patient suffering from a cold. But there were other factors, such as the site of the operation. More pulmonary complications were seen after abdominal than after other operations. This he thought was because the patients did not want to breathe deeply because of pain. Men are more liable than women, because they habitually use the diaphragm less in breathing.

He had used the "iron lung" as a prophylaxis against post-operative pulmonary complications. The patient is introduced to the "iron lung" for one or two hours before operation and is returned to it immediately the operation is over. As much morphine can be given as may be necessary to abolish pain.

The so-called "ether convulsions" he does not believe are due to ether, they can occur with any anæsthetic. The treatment is with barbiturates (nembutal, evipan or pentothal).

Mr. Adams, he thought, painted too rosy a picture of spinal anæsthesia, although there were those who painted the other side too black.

In the Army and the R.A.F. cases of meningism had occurred after the use of percaine. The cases had occurred in a wide variety of places and in many men. Until some explanation of this occurrence of meningism was discovered the use of percaine was stopped. In administering percaine he gives it high.

Headache would occur whatever the technique. He referred to the difficulty of sterilizing the drugs for spinal anæsthesia ; flaws in the glass might render ampoules unsafe.

He was inclined to disagree with Mr. Kindersley in reliance upon colour of the blood. Cyanosis might indicate respiratory obstruction, but nitrous oxide alone will de-oxygenate the blood. An anæmic patient will not show cyanosis, there may be insufficient hæmoglobin to demonstrate anoxia. It was true that the anæsthetist might allow the patient to fluctuate, but sometimes the surgeon might be too violent. The case calls for team-work. If a patient's heart is sound he can stand some anoxia.

At the close of the discussion Group Captain Macintosh demonstrated some apparatus which included the Oxford Inflator and the Oxford Vaporiser. The former can be used in any case of emergency where artificial respiration is necessary. The mask is applied to the patient's face, the airway made clear by pulling the jaw forward. Pressure on a small lever allows the patient's lungs to be inflated intermittently with oxygen for as long as necessary. The Oxford Vaporiser is an apparatus for the administration of the vapour of any liquid anæsthetic in known concentrations. In the form in which it is at present made it is used to volatilize ether, since this is generally considered the anæsthetic with the greatest range of safety. The mask is applied to the patient's face and on inspiration air is drawn over either direct to the patient or can be diverted through the ether chamber. Movement of the control handle determines the amount of the inspired air which passes over the ether, and in this way the ether concentrations delivered to the patient can be predetermined at any desired level.

